# Genetic Diversity of Arabica Coffee (*Coffea arabica* L.) in Nicaragua as Estimated by Simple Sequence Repeat Markers

**DOI:** 10.1100/2012/939820

**Published:** 2012-06-04

**Authors:** Mulatu Geleta, Isabel Herrera, Arnulfo Monzón, Tomas Bryngelsson

**Affiliations:** ^1^Department of Plant Breeding and Biotechnology, Swedish University of Agricultural Sciences, P.O. Box 101, 230 53 Alnarp, Sweden; ^2^Department of Plant Protection, National Agrarian University, P.O. Box 453, Managua, Nicaragua

## Abstract

*Coffea arabica* L. (arabica coffee), the only tetraploid species in the genus *Coffea*, represents the majority of the world's coffee production and has a significant contribution to Nicaragua's economy. The present paper was conducted to determine the genetic diversity of arabica coffee in Nicaragua for its conservation and breeding values. Twenty-six populations that represent eight varieties in Nicaragua were investigated using simple sequence repeat (SSR) markers. A total of 24 alleles were obtained from the 12 loci investigated across 260 individual plants. The total Nei's gene diversity (*H*_*T*_) and the within-population gene diversity (*H*_*S*_) were 0.35 and 0.29, respectively, which is comparable with that previously reported from other countries and regions. Among the varieties, the highest diversity was recorded in the variety Catimor. Analysis of variance (AMOVA) revealed that about 87% of the total genetic variation was found within populations and the remaining 13% differentiate the populations (*F*_*ST*_ = 0.13; *P* < 0.001). The variation among the varieties was also significant. The genetic variation in Nicaraguan coffee is significant enough to be used in the breeding programs, and most of this variation can be conserved through *ex situ* conservation of a low number of populations from each variety.

## 1. Introduction

*Coffea arabica* L. (arabica coffee) is a self-fertile allotetraploid species that belongs to the genus *Coffea* in the family Rubiaceae [1, 2]. Out of the 103 species in the genus, arabica coffee is the only tetraploid species (2*n* = 4*x* = 44), the remaining species being diploid with 2*n* = 2*x* = 22 chromosomes [3]. Arabica coffee originated from a relatively recent hybridization between *Coffea canephora* (robusta coffee) and *C. eugenioides *or their ecotypes in the plateaus of Central Ethiopia [2, 4]. Coffee is mainly grown in tropical and subtropical regions and is an important cash crop in more than 60 countries in South and Central America, Asia, and Africa with an acreage of over 11 million ha [5].

Coffee production is an important economic activity in Central America and accounts for about 10% of the world coffee production [6]. In Nicaragua, large-scale coffee production was started in the 1850s, and since 1870, coffee is the main export crop [7, 8]. Most of the coffee production in the country comes from arabica coffee and the most cultivated varieties are Caturra, Catuai, Bourbon, and Typica [9]. More than 70% of Nicaraguan coffee is produced at elevations between 600 and 1500 m above sea level (asl) in the north central part of the country, where it is considered optimal for coffee production; and the rest is produced below 600 m asl in the south pacific region [10]. During 2010, the total production in the country was about 78 kilo tonnes [11].

Several studies have shown that the genetic diversity of arabica coffee is low when compared to that of robusta coffee [2, 12–19] due to its narrow genetic base associated with autogamy, evolutionary history, and domestication. This narrow genetic base has been reflected in different forms that include the lack of resistant genotypes to various pests and diseases (e.g., [20–22]). The genetic base of arabica coffee in the American content is even narrower, as it represents only a small subset of the genetic variations present within the arabica coffee gene pool [14, 20] and are more prone to various pests and diseases [20, 23, 24]. Thus, enhancement of its resistance to pests and diseases is becoming a crucial priority for economic and sustainable coffee production. This is being done through crossing arabica coffee with other coffee species, particularly robusta coffee [12, 21, 23, 25, 26] and through selection of genotypes of interest from the arabica coffee gene pool [27, 28].

Detecting and quantifying genetic variation in crop species is important for successful conservation of genetic resources and plant breeding. Molecular marker techniques, such as random amplified polymorphic DNA (RAPD), amplified fragment length polymorphism (AFLP), and simple sequence repeats (SSR, also known as microsatellites) have been used for genetic diversity analysis in wild and cultivated coffee [13, 14, 16–19, 29–31]. However, there is little information on the genetic diversity of arabica coffee varieties in Nicaragua. Hence, the present study was conducted to estimate the genetic diversity and population genetic structure of arabica coffee in Nicaragua using SSR markers.

## 2. Materials and Methods

### 2.1. Plant Material and DNA Extraction

Coffee seeds from twenty-six populations representing eight arabica coffee varieties were used in this study. Each population was represented by ten individual coffee trees. Fresh coffee berries were collected between December 2009 and February 2010 from the main coffee growing provinces of Nicaragua (Table 1). The berries were dried up at room temperature and processed to obtain seeds. The seeds were then grown in pots in a greenhouse at a mean temperature of 28°C. Individually sampled leaf tissue from the plants grown in the greenhouse was placed in 2 mL Eppendorf microcentrifuge tubes and immediately frozen in liquid nitrogen and stored at −80°C until DNA extraction. After the frozen samples were milled using a Retsch MM400 shaker (Haan, Germany), DNA was extracted using a modified CTAB procedure, as described in Bekele et al. [32]. DNA quality and concentration was measured using a Nanodrop ND-1000 spectrophotometer (Saveen Werner, Sweden).

### 2.2. SSR-PCR

Twenty-five SSR primer-pairs were initially screened for good amplification, polymorphism, specificity to their target loci, and suitability of the allele size for multiplexing. This led to the selection of twelve primer-pairs for final analysis (Table 2). The forward primers of selected primer-pairs were fluorescently 5′-labeled with either 6FAM, VIC, NED, or PET fluorescent dyes. The reverse primers were PIG-tailed with “*GCTTCT*” to avoid a nontemplated addition of a single nucleotide by *Taq *DNA polymerase to the PCR product, as described in Ballard et al. [34].

The PCR reactions were carried out in a volume of 25 *μ*L containing 25 ng genomic DNA, 0.3 *μ*M forward and reverse primers, 2 mM MgCl_2_, 0.3 mM dNTPs, 1 U *Taq* DNA polymerase (Sigma, Germany), and 1 × PCR buffer (10 mM Tris-HCl, pH 8.3 and 50 mM KCl). The reactions were performed using the GeneAMP PCR system 9700 thermocycler using the following temperature profiles: initial denaturation at 95°C for 3 min, followed by six touchdown cycles of denaturation at 94°C for 30 sec, annealing at *X*-*Y*°C (−1°C/cycle) for 30 sec and extension at 72°C for 45 sec, and then 32 cycles of denaturation at 94°C for 30 sec, annealing at *Y*°C for 30 sec, and extension at 72°C for 45 sec, and a 20 min final extension step at 72°C. The annealing temperature (*T*_*a*_) was changed based on the melting temperature (*T*_*m*_) of each primer-pair (Table 1).

For each locus, amplification was confirmed by running 5 *μ*L of the PCR products on 1.5% ethidium bromide containing agarose gels. The PCR products of the twelve primer-pairs were multiplexed into two panels, each of which containing six PCR products. In each panel, the size difference between the PCR products labeled with the same fluorescent dyes was at least 80 bp to avoid overlapping. The multiplex PCR products were then analyzed using an ABI Prism 3730 DNA Analyzer (Applied Biosystems) at Genomics Core Facility of the University of Gothenburg, Sweden.

### 2.3. Genotyping and Data Analysis

The allele peaks were visually inspected and then analyzed using PEAK SCANNER V1.0 software (Applied Biosystems) based on the internal Genescan-500 LIZ size standard. Each peak was considered as an allele at a codominant locus and the genotype of each individual at each locus was recorded. The Free Tree-Freeware program [35] was used to generate Nei's standard genetic distance and for cluster analysis and bootstrapping. TreeView (Win32) 1.6.6 program [36] was used to view the trees. Analysis of molecular variance (AMOVA) was conducted using Arlequin ver. 3.5-2 [37].

## 3. Results

### 3.1. Total and within-Population Genetic Variation

Out of the 12 loci analyzed, eight loci were polymorphic whereas only one allele was detected across the 260 individuals analyzed in each of the remaining four loci. The four monomorphic loci were *838*, *DCM06*, *Sat235,* and *SSR06* (Table 2). The overall gene diversity for each polymorphic locus varied from 0.01 (*Cam35*) to 0.55 (*SSR09*). In addition to *SSR09*, the other loci with a relatively high level of gene diversity were *CM5*, *Sat207,* and *Cam03* with *H*_*T*_ of 0.50, 0.50 and 0.54, respectively (Table 3).

The total gene diversity (*H*_*T*_) and the within-populations gene diversity (*H*_*S*_), estimated based on Nei's gene diversity [38], were 0.353 and 0.291, respectively (Table 3). The genetic diversity of each population (*H*_Loci_), which is the average gene diversity across the eight polymorphic loci, and the percent polymorphic loci (PPL) were also analyzed. *H*_Loci_ ranged from 0.23 to 0.47, whereas %PL ranged from 0.33 to 0.58 (Table 1). At the variety level, the mean Nei's gene diversity ranged from 0.24 (variety Maracaturra) to 0.37 (variety Catimor) with corresponding lowest and highest %PL of 0.33 and 0.52. Variety Catimor showed the highest gene diversity in five of the eight polymorphic loci (Table 3). The overall mean gene diversity and %PL per population were 0.29 and 0.42, respectively.

**Figure 1:**
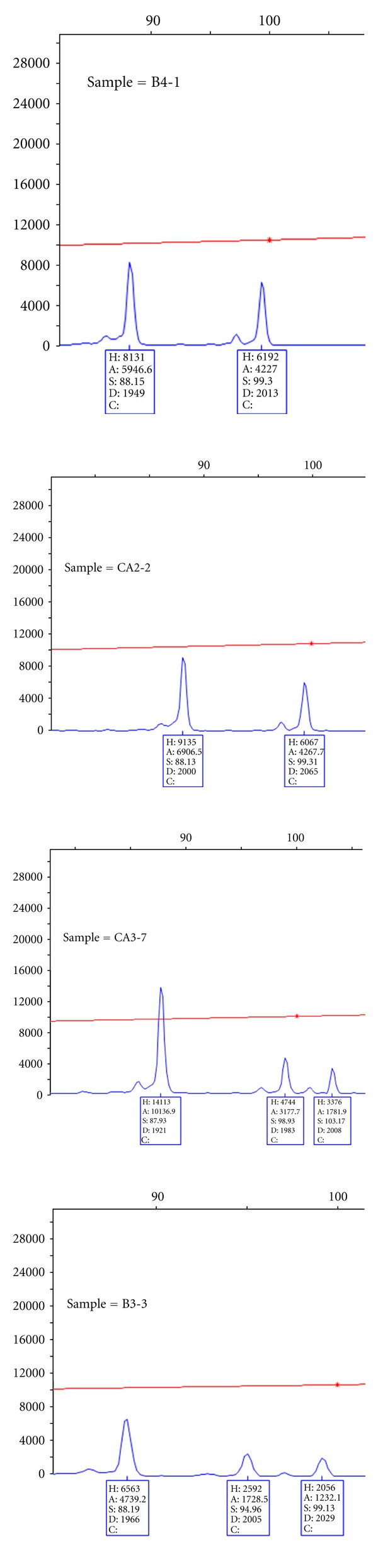
The electrophoretogram showing the alleles at the SSR locus *Sat207*.

Population-specific rare alleles, with frequencies ranging from 0.025 to 0.1, were detected in five of the 26 populations. An 89 bp allele unique to population B3 was detected at locus *Sat207* (Figure 1) at a frequency of 0.025. Similarly, a 97 bp allele was detected in populations CA3 and CM5 at this locus at the same frequency. The other populations bearing unique and rare alleles were *B4* and *CT7*. These populations had unique alleles at loci *SSR03* (frequency = 0.1) and *Cam35* (frequency = 0.025), respectively. 

### 3.2. Genetic Variation among Populations and Groups

The genetic differentiation of populations was estimated based on gene diversity (*G*_*ST*_; [39]) and AMOVA (*F*_*ST*_; [37]). The overall mean *G*_*ST*_ and *F*_*ST*_ were 0.23 and 0.13, respectively (Tables 3 and 4). The estimates of *G*_*ST*_ varied from 0.00 (*CM5*) to 0.62 (*Cam16*) when calculated for each locus. The values of both *G*_*ST*_ and *F*_*ST*_ at locus *CM5* were zero although each population has a gene diversity of 0.5. This is due to the fact that all individuals were heterozygous for the two alleles at this locus. The same is true for locus *Sat207* except that few individuals in three of the 26 populations had additional rare alleles. At these two loci, both the total and within-population gene diversity was high but with no differentiation between the populations.

Overall, AMOVA revealed a highly significant genetic variation among populations (*P* < 0.0001; Table 4) accounting for 13.5% of the total variation. The differentiation among varieties was also significant (*F*_*CT*_ = 0.08; *P* = 0.023) contributing 7.9% to the total genetic variation. The presence of rare alleles in four of the eight varieties contributed to the significant differentiation obtained. On the other hand, AMOVA revealed no significant variation among the two coffee growing regions and among the eight provinces (*P* > 0.4; Table 4). The pairwise AMOVA in the 26 populations revealed that each population was significantly differentiated from at least four populations. The most differentiated populations were CM2, CM3, and CM4, all of which belong to the variety Catimor. Pairwise *F*_*ST*_ showed that CM2 and CM3 were significantly differentiated from each other as well as from all other populations. Population CM4 was significantly differentiated from all populations except from B2 (Table 5). Population CA5 was significantly differentiated from only four populations (B2, CM2, CM3, and CM4). At variety level, Catimor, Catuai rojo, and Pacas were differentiated from each other and all other varieties (Pairwise *F*_*ST*_; Table 6).

### 3.3. Genetic Distance and Cluster Analysis

**Figure 2:**
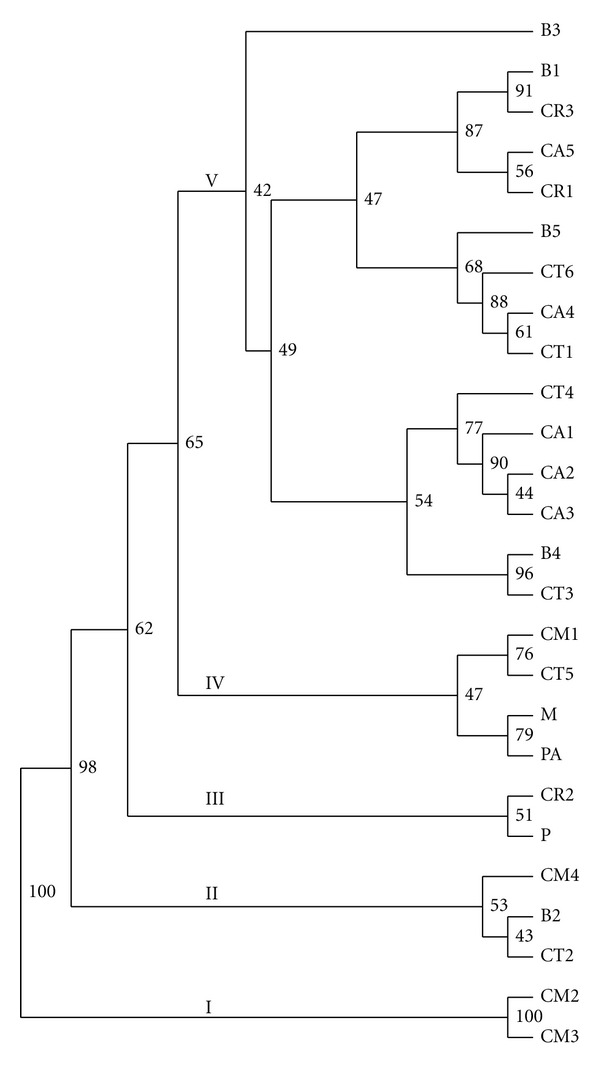
UPGMA phenogram for the 26 coffee populations based on Nei's standard genetic distance. Numbers in front of the branches are bootstrap values.

**Figure 3:**
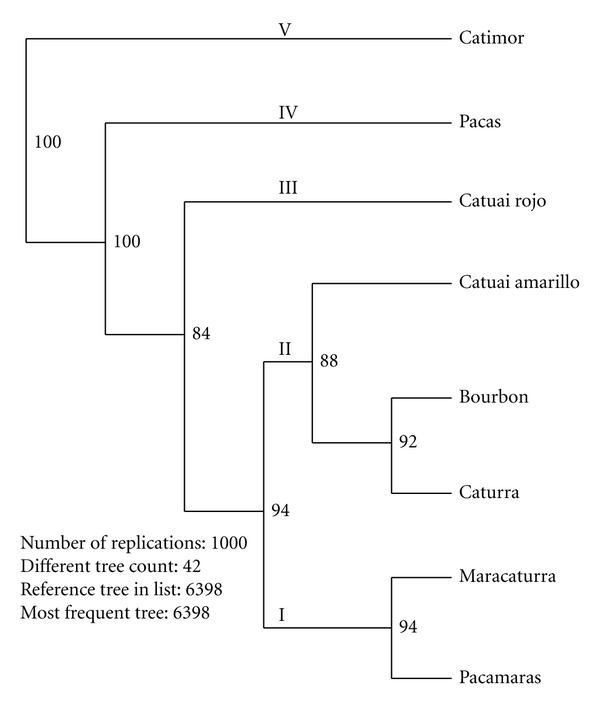
UPGMA phenogram for the eight coffee varieties based on Nei's standard genetic distance. Numbers in front of the branches are bootstrap values.

The Nei's standard genetic distance between populations ranged from less than 0.001 (e.g., CA1 versus CA2) to 0.392 (B3 versus CM2) with the overall mean of 0.060 (Table 5). The genetic distance between the varieties ranged from 0.001 (Caturra versus Bourbon and Caturra versus Catuai amarillo) to 0.121 (Catimor versus Catuai amarillo) with the overall mean of 0.031 (Table 6). The Nei's genetic distance-based cluster analysis revealed five clusters supported by moderate-to-high bootstrap values. Cluster I contained two populations from the variety Catimor (CM2 and CM3) with a bootstrap support of 100%. The 98% bootstrap supported Cluster II contained three populations (CM4, B2, and CT2), which belong to three different varieties. Similarly, cluster III comprised two populations (CR2 and P) from the Catuai-rojo and Pacas varieties with a 62% bootstrap support. Populations CM1, CT5, M, and PA, each of which belongs to different varieties, were placed under Cluster IV with a 65% bootstrap support. Cluster V is the largest cluster comprising 15 populations that were subclustered into three groups. However, the bootstrap support for the subclusters was low. In all clusters except cluster I, populations from more than one variety were clustered together showing a poor clustering of populations according to their varieties (Figure 2). At the variety level, Maracaturra and Pacamaras formed cluster I, whereas Catuai Amarillo, Bourbon, and Caturra formed cluster II with a 94% bootstrap support (Figure 3). The remaining three varieties remained solitary.

## 4. Discussion

### 4.1. The SSR Loci and Alleles

The difference in number of nucleotides between alleles obtained at the polymorphic loci in the present study indicates that the source of polymorphism was mainly the variation in number of repeat motifs of the SSRs. For example, *CAM35* is a hexanucleotide repeat SSR [33], and the size of the alleles obtained in the present study was 207 bp and 213 bp. Similarly, the size of the two alleles of *CM5*, a trinucleotide repeat SSR locus [15], was 91 bp and 94 bp. However, the difference in size of the alleles observed at locus *Sat207* appeared to be due to a combination of differences in the repeat motif and other types of variation, such as indels in the flanking sequences, as the difference in length between the alleles varied from three to four nucleotides (82 bp, 89 bp, 93 bp, 97 bp; Figure 1).

In addition to their application for analysis of genetic diversity, SSR markers have several other applications that include their use as markers for desirable traits. Among the SSRs used in the present study, *Sat207* and *Sat235* were reported to be tightly linked to locus *Ck-1* that carries a major gene conferring resistance to the coffee berry disease (CBD) with *Sat235* more closely linked to the gene than *Sat207* [23]. CBD is a fungal disease caused by *Colletotrichum kahawae* that may cause severe damage in arabica coffee. 

The SSR* Sat235* was monomorphic across the 26 populations, and it is less likely that it can be a useful marker for genetic linkage analysis of *Ck-1* in Nicaraguan coffee. On the other hand, *Sat207* was polymorphic with two major alleles (ca 82 bp and 93 bp) and two rare alleles (ca 89 bp and 97 bp). Taking into consideration the amphidiploid nature of arabica coffee, it is most likely that the 82 bp allele on one hand and the other three alleles on the other hand originated from different progenitor genomes of arabica coffee (Figure 1).

If variation exists at the *Ck-1 *locus in Nicaraguan arabica coffee that gives resistance to CBD, the polymorphism detected at *Sat207* is worth considering during the development of molecular markers linked to the resistance trait. Since arabica coffee is generally considered susceptible to CBD (e.g., [23, 40]), resistant genotypes should be rare and thus it would be interesting to evaluate the genotypes carrying the two rare alleles for resistance to this disease. Developing CBD-resistant arabica coffee varieties through identification of mutants is a method of choice, as it is simple and straight forward as compared to transferring resistance genes from other coffee species that requires crossing with donor genotypes followed by backcrossing to restore desirable traits. Considering that alleles of the same size at locus *sat207 *are identical by decent, it would also be interesting to compare the allele linked to resistance to CBD [23] with the allele introgressed to arabica coffee from robusta coffee [12], as this helps to assign the alleles to the two progenitor genomes.

Another interesting locus to discuss is *CM5*. Two alleles were detected at this locus and all the 260 individual plants studied were heterozygous for the two alleles. Baruah et al. [15] also identified only two alleles at this locus in arabica coffee. Several authors have reported a high cross-species transferability of SSR markers including the EST-SSRs within the genus *Coffea *(e.g., [15, 18, 41]). Given that arabica coffee is autogamous [3], the 100% heterozygosity obtained at this locus can only be explained by its amphidiploid nature. The two alleles should have been originated from different arabica coffee ancestral genomes. Baruah et al. [15] obtained 70% heterozygosity in arabica coffee at this locus, unlike the present study, suggesting that the two arabica coffee genomes of some genotypes carried the same alleles due to homoplasy. At this and other similar loci, fixed heterozygosity is the result when the two homoeologous loci are monomorphic and homozygous within the studied populations. The results clearly suggest the lack of recombination between the chromosomes of the two ancestral genomes due to the amphidiploid nature of arabica coffee.

 Cubry et al. [19] obtained only two alleles per locus in the study that involved sixty SSR loci, and based on this they treated their data as diploid species data. However, a maximum of two alleles per locus is not always the case in arabica coffee, as shown in the present study. For example, three alleles were obtained at locus *CAM03* in most individual plants analyzed. Three alleles per genotype were also observed at locus *Sat207*. These SSRs are reliable evidence that shows the presence of loci bearing nonrecombining alleles in arabica coffee representing the homoeologous loci from the two progenitor genomes. This supports the amphidiploid nature of the allotetraploid arabica coffee previously reported based on cytological evidence [2, 42].

### 4.2. The within- and among-Population Genetic Variation

The narrow genetic base of arabica coffee caused by rigorous selection during domestication and breeding has been reported by several authors (e.g., [14–16, 18, 19]). For example, Cubry et al. [19] reported a mean of 2.1 alleles per locus for arabica coffee, which was the lowest among the *Coffea* species they studied. This is comparable with 2.3 alleles/locus obtained in the present study. Similarly, Moncada and McCouch [16] reported a mean of 1.9 alleles per locus.

In the present study, the mean Nei's total (*H*_*T*_) and within-population (*H*_*S*_) gene diversity were estimated to be 0.35 and 0.29, respectively. The estimates for these parameters were 0.22 and 0.07, in that order, for the RAPD-based study of the Ethiopian arabica coffee by Anthony et al. [13]. Similarly, Cubry et al. [19] reported a mean gene diversity of 0.30 for the arabica coffee material they studied using SSR markers, whereas Aga et al. [43] reported an *H*_*T*_ of 0.37 using ISSR markers in Ethiopian forest coffee. Thus, the level of genetic variation in Nicaraguan arabica coffee is comparable to that previously reported from several countries and regions. The presence of the major SSR alleles across all the populations in very high frequencies in the present study suggests a narrow gene pool of arabica coffee in Nicaragua in line with previous reports. This suggests some difficulties in finding genotypes bearing desirable traits, such as resistance to diseases and pests within the domesticated arabica coffee gene pool.

### 4.3. The Arabica Coffee Varieties in Nicaragua

The cluster analysis of the SSR data for the 26 populations revealed that, in most cases, the clustering pattern of the populations was not in line with their varietal classification. The principal coordinate analysis (PCoA) of the 260 individual plants (data not shown) revealed the presence of divergent genotypes in populations B2, B3, CM3, CT2, CT5, and PA, which partly explains the poor clustering of populations according to their variety of origin. Given that arabica coffee is an autogamous species; such a poor clustering pattern of populations according to variety of origin is somewhat unexpected. However, the processes through which these varieties were developed may partly explain the lack of a clear differentiation between the populations of the different varieties. For example, the variety Caturra was developed from mutant genotypes of the variety Bourbon (http://www.coffeeresearch.org/coffee/varietals.htm). A significant differentiation between these two varieties may not be expected due to the relatively short time elapsed since the development of the variety Bourbon, especially at selectively neutral loci that include most of the SSRs used in the present study. Similarly, Catuai was the result of a cross between Mundo Novo and Caturra and thus there may not be a clear genetic differentiation between the Caturra and Catuai varieties at this stage. A relatively close relationship between the Bourbon, Caturra, and Catuai varieties can be observed from Figure 3. However, other possible factors, such as some degree of gene flow between varieties through cross pollination, might have also contributed to the population genetic structure obtained.

Among the eight coffee varieties we studied, the highest genetic diversity was recorded in the variety Catimor (GD = 0.37 and PPL = 0.52). This variety is interesting not only because of its high genetic diversity but also because two of its populations (CM2 and CM3) were significantly differentiated from all the other populations (Table 5). At locus *471*, the alleles recorded in CM2 and CM3 were different from those in the other populations, excluding CM4. The relatively high diversity in Catimor can be partly explained by the fact that it was the result of a cross between the variety Caturra of arabica coffee and the Timor hybrid, which is a natural hybrid between arabica and robusta coffee [21, 23]. Robusta coffee has been reported to have a relatively high genetic diversity compared to arabica coffee in several studies (e.g., [16, 19]). Thus, a wise use of the genetic diversity in the locally adapted populations of the variety Catimor in coffee breeding programs in Nicaragua is very important. The presence of allelic variation at several loci in the other coffee varieties suggests the significance of using the existing genetic variation in these varieties in the hybrid breeding program to develop superior and improved varieties.

Root-knot Nematodes (RKN) of the genus *Meloidogyne* cause major damage in coffee worldwide, and coffee breeding for durable resistance to RKN is now a major goal in coffee producing countries [28]. *Meloidogyne exigua* and *M. incognita *are known RKN attacking arabica coffee in Nicaragua [44]. The best method to reduce the damage caused by RKN in coffee is through developing resistant varieties. Bertrand et al. [45] reported resistant arabica coffee genotypes to *M. arabicida* in Costa Rica, which suggests that identification of resistant genotypes to *M. exigua* and *M. incognita *from arabica coffee in Nicaragua may be possible. In addition, the resistance gene *Mex-1* identified in robusta coffee and successfully introgressed into arabica coffee by Noir et al. [25] suggests the need to give attention to arabica coffee varieties developed through hybridization of the two cultivated *Coffea* species, such as the variety Catimor for pest and disease resistance.

Overall, the level of genetic diversity of arabica coffee in Nicaragua is generally low and is comparable to that previously reported for arabica coffee from other countries and regions. Therefore, it should be promoted through crossing with other closely related species such as robusta coffee. In addition, the presence of rare alleles in some populations suggests the need to explore such populations in order to identify mutants bearing desirable traits. The significant differentiation between most Nicaraguan arabica coffee varieties suggests that varieties grown in the country should be analyzed for resistance/tolerance to major biotic and abiotic stresses. On the other hand, the absence of a significant differentiation between the coffee populations based on regions of origin suggests that germplasm collecting missions should prioritize the representation of coffee varieties over coffee growing regions in the country.

## Figures and Tables

**Table 1:** List of 26 arabica coffee populations used in this study representing eight coffee varieties in Nicaragua, and their geographic descriptions and SSR-based estimates of gene diversity (*H*_Loci_) and percent polymorphic loci (PPL).

Population/variety	Province	Position		*H*_Loci_^a^	PPL*	Population/variety	Province	Position		*H*_Loci_^a^	PPL
B1	Masatepe	N 11°54′	W 86°08′	0.26	0.42	CA1	El Crucero	N 12°00′	W 86°17′	0.24	0.42
B2^b^	Jinotepe	N 11°50′	W 86°12′	0.47	0.58	CA2	Masatepe	N 11°54′	W 86°07′	0.24	0.42
B3^c^	Dipilto	N 13°45′	W 86°30′	0.26	0.42	CA3^e^	Matagalpa	N 13°10′	W 85°29′	0.24	0.33
B4	Jinotega	N 13°10′	W 85°51′	0.25	0.42	CA4	Dipilto	N 13°45′	W 86°47′	0.27	0.33
B5	Quilalí	N 13°36′	W 85°56′	0.28	0.50	CA5	Sn Juan Río Coco	N 13°28′	W 86°09′	0.27	0.33
**Bourbon (B)**				**0.30**	**0.47**	**Catuai amarillo (CM)**				**0.25**	**0.35**
CT1	Masatepe	N 11°53′	W 86°08′	0.28	0.50	CM1	Dipilto	N 13°45′	W 86°32′	0.32	0.50
CT2	Jinotepe	N 11°50′	W 86°12′	0.40	0.58	CM2	Jinotega	N 13°13′	W 85°54′	0.33	0.42
CT3	Matagalpa	N 13°10′	W 85°45′	0.25	0.42	CM3	Quilalí	N 13°36′	W 85°56′	0.43	0.58
CT4	Dipilto	N 13°45′	W 86°30′	0.23	0.33	CM4^f^	Sn Juan Río Coco	N 13°34′	W 86°11′	0.40	0.58
CT5	Jinotega	N 13°13′	W85°52′	0.32	0.58	**Catimor**				**0.37**	**0.52**
CT6^d^	Quilalí	N 13°37′	W 85°58′	0.27	0.42	M	Dipilto	N 13°45′	W 86°29′	0.24	0.33
**Caturra (CT)**				**0.29**	**0.47**	**Maracaturra**				**0.24**	**0.33**
CR1	Matagalpa	N 13°09′	W 85°44′	0.25	0.33	P	Jinotega	N 13°11′	W 85°51′	0.30	0.50
CR2	Dipilto	N 13°45′	W 86°32′	0.24	0.42	**Pacas**				**0.30**	**0.50**
CR3	Sn Juan Río Coco	N 13°29′	W 86°10′	0.26	0.42	PA	Dipilto	N 13°45′	W 86°32′	0.27	0.50
**Catuai rojo (CR)**				**0.25**	**0.39**	**Pacamaras**				**0.27**	**0.50**

^a^Calculated based on the eight polymorphic loci; ^b^population B2 has a unique allele at the locus *Cam35*; ^c^population B3 has a unique allele at the locus *Sat207*; ^d^population CT6 has a unique allele at the locus *SSR03*; ^e^population CA3 has a unique allele at the locus *Sat207*; ^f^population CM4 has a unique allele at the locus *Sat207*. Note: variety names and their corresponding mean genetic diversity values are shown in bold; populations listed above each variety belong to that variety; the overall mean *H*_Loci_ and PPL across all populations and loci were 0.29 and 0.42, respectively.

**Table 2:** List of primer-pairs used to amplify the SSR loci used in this study and their annealing temperature: the repeat motifs of the loci and observed fragment sizes of the alleles.

Locus name	Repeat motif	Primer sequence	Annealing temperature		Observed allele size (bp)
			*X*°C	*Y*°C	
*471* ^ a^	CT	F: TTACCTCCCGGCCAGAC	60	54	292, 318, 320
		R: CAGGAGACCAAGACCTTAGCA			
*838* ^ a^	AC	F: CCCGTTGCCATCCTTACTTA	57	61	97
		R: ATACCCGATACATTTGGATACTCG			
*CaM03* ^ b^	AC	F: CGCGCTTGCTCCCTCTGTCTCT	68	62	188, 194
		R: TGGGGGAGGGGCGGTGTT			
*CaM16* ^ b^	TC	F: AAGGCAGCTGAAGCGGGACAAA	68	62	188, 194
		R: TGGGGAGAGCTGCAGTTGGAGG			
*CaM35* ^ b^	TGGAAG	F: CGAGCTAGAATGGATGACTTGGTTGG	65	59	207, 213
		R: ATACCCGATACATTTGGATACTCG			
*CM5* ^ c^	CCT	F: GTAACCACCACCTCCTCTGC	60	54	185, 188
		R:TGGAGGTAACGGAAGCTCTG			
*DCM06* ^ d^	(T) (TTC)	F: GTAGTCGGTGGGCTTGTGTT	60	54	213
		R**: ** AACGCGGACTAATTGAGGAA			
*Sat207* ^ e^	ng	F: GAAGCCGTTTCAAGCC	57	51	82, 89, 93, 97
		R**: ** CAATCTCTTTCCGATGCTCT			
*Sat235* ^ e^	ng	F:TCGTTCTGTCATTAAATCGTCAA	60	54	167
		R: GCAAATCATGAAAATAGTTGGTG			
*SSR03* ^ d^	TC	F: GGACAAAACACCGCCCAAAATA	62	56	142, 148
		R: AGCGAGACAGAGGAAGGGAATATT			
*SSR06* ^ d^	AAAGG	F: CAGGCACAGAAGGAATGAAGAGC	62	56	126
		R: TGGTGGTATGGAAAACAGGAAGG			
*SSR09* ^ d^	GT	F: TTGGCTTTTGTCCCTCCTTCCTCTG	62	56	124, 126, 130
		R: AGCCCATTTCCCTCTCATCATTTCAAG			

ng: not given in the original reference. References: ^a^: Cubry et al. 2008 [19]; ^b^: Hendre et al. 2008 [33]; ^c^: Baruah et al. 2003 [15]; ^d^: Aggarwal et al. 2007 [18]; ^e^: Gichuru et al. 2008 [23].

**Table 3:** The partitioning of total gene diversity into within and among variety components for eight polymorphic SSR loci, and the number of alleles observed at each locus.

Variety	H_SSR03_	H_CM5_	H_CaM35_	H_Sat207_	H_CaM16_	H_CaM03_	H_471_	H_SSR09_	Overall
Bourbon	0.136	0.500	0.036	0.501	0.075	0.516	0.166	0.500	0.304
Catuai amarillo	0.142	0.500	0.000	0.504	0.000	0.499	0.000	0.384	0.254
Catimor	0.241	0.500	0.000	0.506	0.224	0.538	0.387	0.556	0.369
Catuai rojo	0.048	0.500	0.000	0.500	0.000	0.498	0.000	0.433	0.247
Caturra	0.136	0.500	0.000	0.500	0.060	0.525	0.140	0.468	0.291
Maracaturra	0.000	0.500	0.000	0.500	0.000	0.420	0.000	0.480	0.238
Pacas	0.139	0.500	0.000	0.500	0.000	0.420	0.465	0.375	0.300
Pacamaras	0.049	0.500	0.000	0.500	0.180	0.480	0.000	0.480	0.274
*H_S_*	0.136	0.500	0.007	0.502	0.070	0.509	0.141	0.465	0.291
*H_T_*	0.229	0.500	0.008	0.503	0.186	0.536	0.310	0.550	0.353
*G_ST_*	0.409	0.000	0.091	0.002	0.624	0.051	0.544	0.154	0.234
NA	2	2	2	4	2	2	3	3	2.5 (2.0)*

*The mean number of alleles for the eight polymorphic loci and for all loci including monomorphic ones is 2.5 and 2.0, respectively.

**Table 4:** SSR-based AMOVA for the 26 populations of arabica coffee: (A) without grouping the populations, (B) by grouping the populations according to varieties, (C) by grouping the populations according to regions of origin, and (D) by grouping the populations according to province of origin.

Groups	Sources of variation	Degrees of freedom	Variance components	Percentage of variation	Fixation indices	*P*-value
(A) without grouping populations	AP	25	Va = 0.194	13.50	*F*_*ST*_ = 0.130	Va and *F*_*ST*_ = 0.000
	WP	494	Vb = 1.25	86.50		
	Total	519	1.44			

(B) Populations grouped by varieties	AV	7	Va = 0.115	7.90	*F*_*ST*_ = 0.140	Vc and *F*_*ST*_ = 0.000
	APWV	18	Vb = 0.095	6.50	*F*_*SC*_ = 0.070	Vb and *F*_*SC*_ = 0.000
	WP	494	Vc = 1.25	85.60	*F*_*CT*_ = 0.08	Va and *F*_*CT*_ = 0.023
	Total	519	1.46			

(C) Populations grouped by regions of origin	AR	1	Va = −0.022	−1.60	*F*_*ST*_ = 0.125	Vc and *F*_*ST*_ = 0.000
	APWR	24	Vb = 0.25	14.10	*F*_*SC*_ = 0.139	Vb and *F*_*SC*_ = 0.000
	WP	494	Vc = 1.25	87.50	*F*_*CT*_ = −0.016	Va and *F*_*CT*_ = 0.812
	Total	519	1.43			

(D) Populations grouped by province of origin	APr	7	Va = −0.002	−0.14	*F*_*ST*_ = 0.134	Vc and *F*_*ST*_ = 0.000
	APWPr	18	Vb = 0.196	13.57	*F*_*SC*_ = 0.136	Vb and *F*_*SC*_ = 0.000
	WP	494	Vc = 1.25	86.57	*F*_*CT*_ = −0.001	Va and *F*_*CT*_ = 0.419
	Total	519	1.44			

AP: among populations; WP: within populations; AV: among varieties; APWV: among populations within varieties; AR: among regions; APWR: among populations within regions; APr: among provinces; APWPr: among populations within provinces.

**Table 5:** The Nei's standard genetic distance (above the diagonal) and the pairwise *F*_*ST*_ (below the diagonal) between the 26 pairs of arabica coffee populations.

	B1	B2	B3	B4	B5	CA1	CA2	CA3	CA4	CA5	CM1	CM2	CM3	CM4	CR1	CR2	CR3	CT1	CT2	CT3	CT4	CT5	CT6	M	P	PA
B1		0.065	0.006	0.005	0.005	0.013	0.013	0.014	0.001	0.001	0.010	0.376	0.255	0.093	0.001	0.006	0.000	0.002	0.018	0.006	0.012	0.008	0.002	0.006	0.015	0.004
B2	+		0.073	0.069	0.056	0.074	0.074	0.076	0.064	0.063	0.060	0.156	0.077	0.014	0.067	0.072	0.065	0.058	0.017	0.070	0.077	0.051	0.066	0.070	0.042	0.064
B3	−	+		0.002	0.003	0.004	0.005	0.005	0.004	0.007	0.010	0.392	0.275	0.112	0.009	0.023	0.004	0.004	0.023	0.002	0.004	0.006	0.003	0.006	0.030	0.004
B4	−	+	−		0.001	0.002	0.002	0.003	0.002	0.006	0.008	0.379	0.267	0.110	0.008	0.023	0.003	0.002	0.021	0.000	0.002	0.004	0.001	0.005	0.030	0.003
B5	−	+	−	−		0.003	0.003	0.004	0.002	0.005	0.008	0.350	0.242	0.092	0.008	0.021	0.003	0.001	0.014	0.001	0.003	0.003	0.001	0.005	0.024	0.003
CA1	+	+	−	−	−		0.000	0.000	0.007	0.013	0.011	0.373	0.272	0.124	0.017	0.037	0.010	0.007	0.026	0.002	0.001	0.006	0.004	0.009	0.043	0.007
CA2	−	+	−	−	−	−		0.000	0.006	0.013	0.011	0.373	0.272	0.124	0.017	0.036	0.009	0.006	0.026	0.001	0.001	0.006	0.004	0.009	0.042	0.006
CA3	+	+	+	−	−	−	−		0.008	0.015	0.012	0.375	0.275	0.127	0.019	0.040	0.011	0.008	0.028	0.002	0.001	0.006	0.005	0.010	0.045	0.007
CA4	−	+	−	−	−	+	−	+		0.001	0.007	0.364	0.252	0.098	0.003	0.012	0.001	0.000	0.017	0.003	0.007	0.004	0.000	0.005	0.020	0.003
CA5	−	+	−	−	−	−	−	−	−		0.008	0.360	0.246	0.091	0.001	0.007	0.001	0.002	0.017	0.007	0.013	0.007	0.003	0.005	0.013	0.004
CM1	−	+	−	−	−	−	−	+	+	−		0.297	0.209	0.100	0.011	0.024	0.009	0.008	0.019	0.008	0.011	0.002	0.007	0.006	0.026	0.003
CM2	+	+	+	+	+	+	+	+	+	+	+		0.023	0.191	0.379	0.379	0.376	0.353	0.248	0.378	0.389	0.306	0.369	0.369	0.311	0.345
CM3	+	+	+	+	+	+	+	+	+	+	+	+		0.092	0.255	0.250	0.257	0.243	0.147	0.267	0.280	0.213	0.257	0.259	0.196	0.240
CM4	+	−	+	+	+	+	+	+	+	+	+	+	+		0.090	0.085	0.096	0.091	0.036	0.112	0.124	0.089	0.103	0.101	0.044	0.100
CR1	−	+	−	−	−	+	−	+	−	−	−	+	+	+		0.004	0.001	0.003	0.019	0.009	0.016	0.010	0.005	0.006	0.012	0.005
CR2	−	+	+	+	+	+	+	+	+	−	+	+	+	+	−		0.009	0.013	0.026	0.025	0.037	0.024	0.016	0.020	0.011	0.018
CR3	−	+	−	−	−	+	−	+	−	−	−	+	+	+	−	+		0.001	0.018	0.004	0.009	0.006	0.001	0.005	0.018	0.003
CT1	−	+	−	−	−	−	−	−	−	−	−	+	+	+	−	+	−		0.015	0.003	0.007	0.004	0.001	0.005	0.019	0.003
CT2	+	−	+	+	−	+	+	+	+	−	−	+	+	+	+	+	+	−		0.021	0.027	0.013	0.018	0.020	0.014	0.018
CT3	+	+	−	−	−	−	−	−	−	−	−	+	+	+	−	+	−	−	+		0.001	0.003	0.001	0.005	0.032	0.003
CT4	+	+	−	−	−	−	−	−	+	−	−	+	+	+	+	+	−	−	+	−		0.006	0.004	0.007	0.042	0.006
CT5	−	+	−	−	−	−	−	+	+	−	−	+	+	+	+	+	−	−	−	−	−		0.004	0.004	0.023	0.002
CT6	+	+	−	−	−	+	−	+	+	−	−	+	+	+	+	+	+	−	+	−	+	−		0.004	0.024	0.002
M	−	+	−	−	−	+	−	+	+	−	−	+	+	+	−	+	−	−	+	−	−	−	+		0.020	0.002
P	−	+	+	+	+	+	+	+	+	−	+	+	+	+	−	−	+	+	−	+	+	+	+	+		0.022
PA	−	+	−	−	−	−	−	−	−	−	−	+	+	+	−	+	−	−	−	−	−	−	−	−	+	

+: Significant differentiation between the pair of populations (*P* < 0.05).

−: No significant differentiation between the pair of populations (*P* > 0.05).

**Table 6:** The Nei's standard genetic distance (above the diagonal) and the pairwise *F*_*ST*_ (below the diagonal) between the eight arabica coffee varieties.

	Bourbon	Catuai amarillo	Catimor	Catuai rojo	Caturra	Maracaturra	Pacas	Pacamaras
Bourbon		0.004	0.091	0.007	0.001	0.007	0.017	0.004
Catuai amarillo	+		0.121	0.011	0.001	0.005	0.030	0.003
Catimor	+	+		0.117	0.102	0.119	0.083	0.108
Catuai rojo	+	+	+		0.008	0.009	0.012	0.007
Caturra	−	−	+	+		0.004	0.022	0.002
Maracaturra	−	−	+	+	−		0.020	0.002
Pacas	+	+	+	+	+	+		0.022
Pacamaras	−	−	+	+	−	−	+	

+: Significant differentiation between the pair of populations (*P* < 0.05).

−: No significant differentiation between the pair of populations (*P* > 0.05).
